# A Secure Image Encryption Scheme Based on a New Hyperchaotic System and 2D Compressed Sensing

**DOI:** 10.3390/e26070603

**Published:** 2024-07-16

**Authors:** Muou Liu, Chongyang Ning, Congxu Zhu

**Affiliations:** 1 College of Electronic Information and Physics, Central South University of Forestry and Technology, Changsha 410004, China; moliu@csuft.edu.cn; 2 School of Computer Science and Engineering, Central South University, Changsha 410083, China

**Keywords:** hyperchaotic map, image encryption, compressed sensing, chaotic system

## Abstract

In insecure communication environments where the communication bandwidth is limited, important image data must be compressed and encrypted for transmission. However, existing image compression and encryption algorithms suffer from poor image reconstruction quality and insufficient image encryption security. To address these problems, this paper proposes an image-compression and encryption scheme based on a newly designed hyperchaotic system and two-dimensional compressed sensing (2DCS) technique. In this paper, the chaotic performance of this hyperchaotic system is verified by bifurcation diagrams, Lyapunov diagrams, approximate entropy, and permutation entropy, which have certain advantages over the traditional 2D chaotic system. The new 2D chaotic system as a pseudo-random number generator can completely pass all the test items of NIST. Meanwhile, this paper improves on the existing 2D projected gradient (2DPG) algorithm, which improves the quality of image compression and reconstruction, and can effectively reduce the transmission pressure of image data confidential communication. In addition, a new image encryption algorithm is designed for the new 2D chaotic system, and the security of the algorithm is verified by experiments such as key space size analysis and encrypted image information entropy.

## 1. Introduction

With the popularity of the application of artificial intelligence techniques such as deep learning in the field of computer vision, it has brought about almost revolutionary changes in various industries. In the machine learning of computer vision deep neural network model, the role of the image as the “raw material” for model training is becoming more and more obvious, and a high-performance visual deep neural network model cannot be separated from a good neural network architecture, but also from the support of a large number of image data sets. Not only in the field of artificial intelligence, image data have a wide range of applications in the fields of healthcare, military warfare, transportation, social media, the economy, and finance. With the rapid development of the Internet of Things industry and the application of communication technologies such as 5G, the secure storage and transmission of image data also pose a challenge to the security of the database and communication channel, which is still a relatively basic network security issue [1].

As the research on AI visual models continues to deepen, researchers pay more and more attention to the security of image data. Recently, a number of schemes on image security protection have been proposed [2,3,4], in which image encryption algorithms based on chaotic systems have received wide attention [5,6,7,8,9]. Chaotic systems are particularly suitable as generators of key sequences in cryptographic algorithms due to their high sensitivity to the initial values. A chaotic system is usually a mathematical model divided into a system of continuous time differential equations and a system of discrete time iterative mappings. In general, continuous time differential equation systems can only exhibit chaotic states in three or more dimensions, while discrete time iterative mapping systems can exhibit chaotic states in one dimension. Usually, the higher dimension of a chaotic system means the stronger its chaotic performance; however, the time overhead of generating random key sequences for high-dimensional chaotic systems is large, which is very unfavorable for application scenarios that require real-time encryption. At the same time, the confidential transmission of large amounts of image data also brings large transmission latency. Therefore, chaotic encryption schemes combined with compressed sensing (CS) [10,11,12] have received more and more attention from researchers [13,14,15].

Compressed sensing (CS) is an algorithm in the field of communication that can recover the original signal in signals acquired at a sampling rate much lower than that required by the Nyquist–Shannon Sampling Theorem, which is advantageous for compressing sparse signals like images. In recent years, a number of image-encryption algorithms combining compressed sensing have been proposed. Wei et al. [13] proposed a compression–encryption algorithm combining compressed sensing and optical encryption. Chai et al. [16] proposed an image-encryption scheme based on multi-objective optimization and Block Compressed Sensing (BCS), which improves security and efficiency. Fan et al. [17] proposed a new four-dimensional chaotic system and semi-tensor product compressed sensing model for image data security protection in wireless media sensor networks (WMSNs). Zhang et al. [18] utilized two-dimensional compressed sensing (2DCS) and developed an iterative singular-value thresholding (ISVT) scheme.

Although some existing compression–encryption schemes based on chaotic systems can achieve image security protection, they also have problems such as the narrow parameter intervals of chaotic behaviors, a small key space, and the insufficient quality of images after image decryption and reconstruction. Therefore, it is necessary to construct a chaotic system with better chaotic performance and apply this system to image compression and encryption. The main contributions of this paper are as follows:

(1) A new 2D chaotic system is proposed, which has strong chaotic performance and is advantageous in the construction of measurement matrices and image-encryption algorithms for 2DCS.

(2) Improvements are made on the basis of the two-dimensional projected gradient (2DPG) algorithm [19] to improve the image compression and reconstruction performance.

(3) A new image encryption algorithm is designed, and the security of the encryption algorithm is proven experimentally.

The paper is organized as follows. In Section 2, a new 2D hyperchaotic system is proposed and its chaotic performance is extensively analyzed. An image-compression and -reconstruction scheme based on an improved 2D projected gradient (2DPG) algorithm is presented in Section 3. The newly designed image-encryption and -decryption algorithms are presented in Section 4. The experimental results of the proposed algorithm of this paper are shown and the results are analyzed in Section 5. Finally, the conclusion of this paper is given in Section 6.

## 2. The Newly Designed Hyperchaotic Map

This section introduces the new 2D hyperchaotic system mathematical model and gives a detailed dynamical analysis of the hyperchaotic system model. Next, the bifurcation diagram, Lyapunov exponent (LE), approximate entropy (ApEn), and permutation entropy (PeEn) are introduced. These complex properties show that the hyperchaotic system has good potential for generating random numbers. Therefore, it is suitable for 2D compressed sensing measurement matrix generation and image encryption.

### 2.1. Mathematic Model of the 2D Hyperchaotic Map

The proposed 2D hyperchaotic mapping mathematical model is composed of two trigonometric functions. Its mathematical equation is as follows:

(1) $$\left\{\begin{array}{c} x_{n+1}=\sin^{2}\left(a\pi x_{n}+by_{n}\right)\\ y_{n+1}=\cos^{2}\left(b\pi /y_{n}+ax_{n}\right) \end{array}\right.$$

It can be seen from system (1) that *a* and *b* are the controlling parameters of the hyperchaotic system. The state variables at the *n*-th and n+1-th discrete time points in the iterative Equation (1), respectively, are represented by $x_{n}$ and $x_{n+1}$. Since system (1) consists of trigonometric functions, the following conclusions can be drawn from the properties of trigonometric functions. If $x_{n}\in R$ and $y_{n}\in R$, then $x_{n+1}\in R$ and $y_{n+1}\in R$. System (1) is a mapping of R→R.

### 2.2. Bifurcation Diagram and Phase Diagram

**Figure 1:**
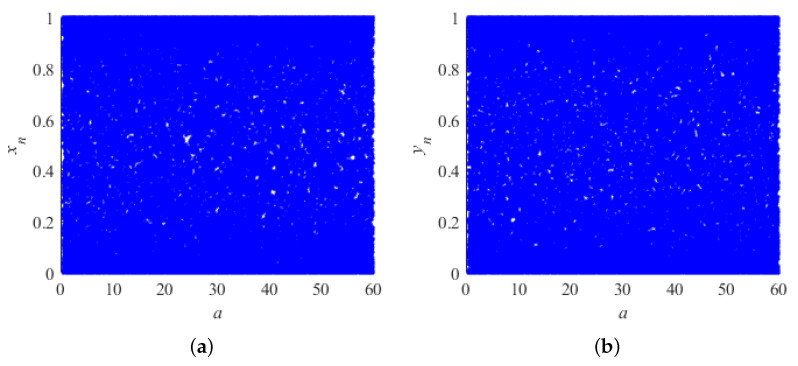
The bifurcation diagram of system (1): (**a**) bifurcation diagram of different parameters a corresponding to variable x; (**b**) bifurcation diagram of different parameters a corresponding to variable y.

The bifurcation diagram visualizes the range of parameters that characterize the chaotic properties of system (1). The values of the state variables derived iteratively from a well-performing chaotic system should present a chaotic state within their range of values. This is shown in Figure 1 by the fact that the values of the state variables $x_{n}$ and $y_{n}$ are spread over the entire interval [0,1] for different ranges of control parameters. It shows that the two-dimensional system is ergodic and satisfies the characteristics of chaos. The bifurcation diagrams for the state variables $x_{n}$ and $y_{n}$ obtained by controlling the variable parameters *a* in the range [0,60] and b=30 are shown in Figure 1. The bifurcation diagram characterization for parameter *b* is similar to the following figure and will not be repeated here.

**Figure 2:**
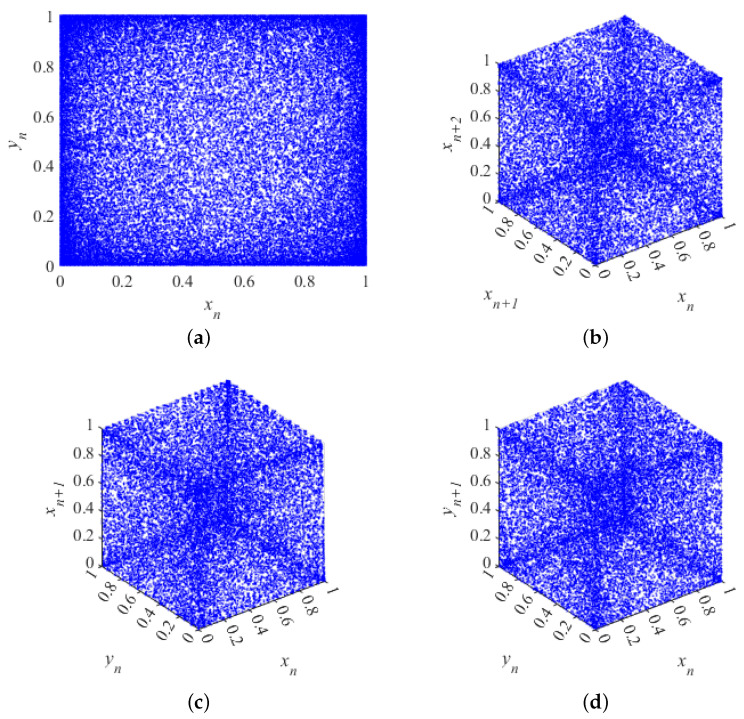
Phase diagram of system (1): (**a**) 2D attractor when $\left(x_{0},y_{0}\right)=\left(0.2,0.3\right)$; (**b**) the relationship between the three iterative sequences $x_{n},x_{n+1},x_{n+2}$; (**c**) the relationship between the three iterative sequences $x_{n},y_{n},x_{n+1}$; (**d**) the relationship between the three iterative sequences $x_{n},y_{n},y_{n+1}$.

The phase diagram for hyperchaotic systems is a graphical tool used to visualize the relationship between the state variables of a system in a nonlinear dynamical system. In hyperchaotic systems, where the system has multiple iterative equations, the phase diagram usually shows the interactions of at least three state variables, since a two-dimensional phase diagram cannot adequately describe the complexity of hyperchaotic systems. The phase diagram is a graphical representation used to show the evolution of the state of a dynamic system over time. In a phase diagram, one or more variables of the system serve as axes, and changes in another variable or variables are plotted on these axes. For hyperchaotic systems, phase diagrams can show the interactions and dynamics between multiple variables. The phase diagram of system (1) with initial values $\left(x_{0},y_{0}\right)=\left(0.2,0.3\right)$ and control parameters a=20,b=40 is shown in Figure 2.

As can be seen from the figure, the hyperchaotic system (1) has a complex trajectory. It shows that the chaotic system proposed in this paper has a high degree of state value randomness and is suitable as a generator of pseudo-random sequences.

### 2.3. Lyapunov Exponent

The Lyapunov exponent (LE) curve for chaotic systems is a metric used to quantify the degree of chaos in a system. The Lyapunov exponent describes the sensitivity of the trajectory of the system, i.e., how small differences in the initial conditions evolve over time. A positive Lyapunov exponent indicates that the system is chaotic. If two or more positive Lyapunov exponents exist for a chaotic system, the system is hyperchaotic, which indicates a higher degree of randomness and more complex dynamical behavior than a normal chaotic system. In order to calculate the Lyapunov exponent for a two-dimensional hyperchaotic system, the following method can be used [20].

Suppose a two-dimensional hyperchaotic mapping is represented by the following equation:

(2) $$f\left(x,y\right)=\left\{\begin{array}{c} x_{i+1}=f_{1}\left(x_{i},y_{i}\right)\\ y_{i+1}=f_{2}\left(x_{i},y_{i}\right) \end{array}\right.$$

The Jacobian matrix of the 2D hyperchaotic mapping can be obtained from Equation (2).

(3) $$J\left(x_{i},y_{i}\right)=\left(\begin{array}{cc} \frac{\partial f_{1}\left(x,y\right)}{\partial x} & \frac{\partial f_{1}\left(x,y\right)}{\partial y}\\ \frac{\partial f_{2}\left(x,y\right)}{\partial x} & \frac{\partial f_{2}\left(x,y\right)}{\partial y} \end{array}\right)$$

The eigenvalues of the matrix $J(x_{i},y_{i})$ are computed to be $\lambda_{1}\left(J\right)$ and $\lambda_{2}\left(J\right)$, respectively. From these two eigenvalues, the Lyapunov exponent of system (2) can be calculated using the following equation:

(4) $$LE_{i}=\lim_{N\to \infty }\frac{1}{N}\sum_{i=1}^{N-1}\ln\left|\left.\lambda_{i}\left(J\right)\right|\right.$$

where N denotes the number of iterative rounds to iteratively obtain the chaotic sequence.

**Figure 3:**
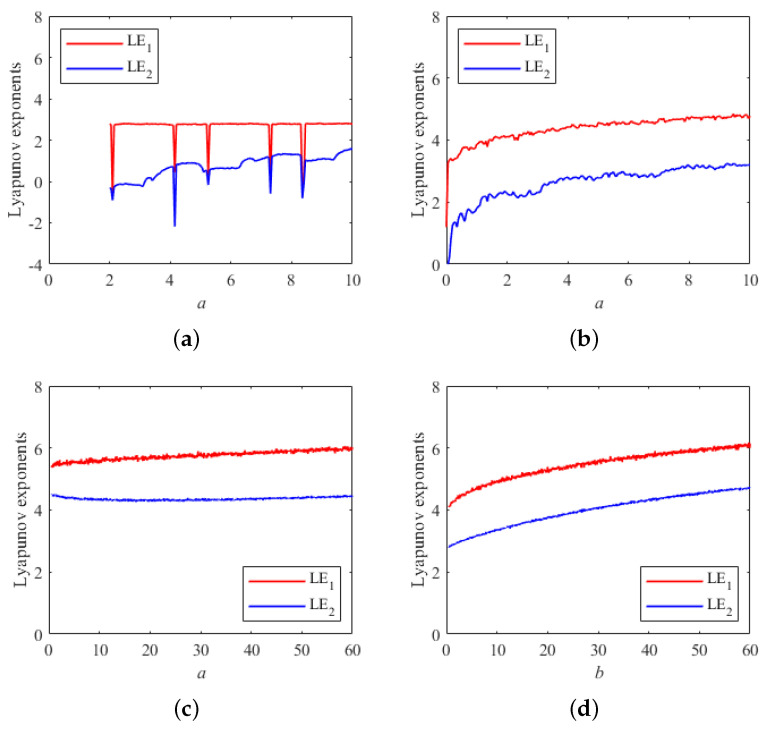
Graphs of Lyapunov exponents for 3 different 2D chaotic systems: (**a**) plot of Lyapunov exponent for 2D-CSCM; (**b**) plot of Lyapunov exponent for 2D-CLII; (**c**) plot of Lyapunov exponent for the proposed map corresponding to parameter *a*; (**d**) plot of Lyapunov exponent for the proposed map corresponding to parameter *b*.

By comparing the magnitude of the LE values of different chaotic systems, it is possible to infer the degree of randomness of the sequences generated by different systems. Figure 3 shows the LE plots of the recently proposed 2D-CSCM map [21], the 2D-CLII map [22], and the new 2D map proposed in this paper. Both the 2D-CSCM map and the 2D-CLII map have only one control parameter *a*. As can be seen from Figure 3a, the Lyapunov exponential curves of 2D-CSCM are less stable, and the LE values corresponding to individual parameters are even less than zero. From Figure 3b, it can be seen that there are two positive Lyapunov exponential curves for 2D-CLII when the control parameter *a* is varied in the interval [0,10], but they are both smaller than the 2D map proposed in this paper. Figure 3c shows the variation of the Lyapunov exponent with respect to the parameter *b* = 40 while a varies between 0 and 60. Figure 3d shows the variation of the Lyapunov exponent with respect to the parameter *a* = 30 while *b* varies between 0 and 60. As can be seen from Figure 3, the 2D system proposed in this paper has two positive Lyapunov exponents, proving that the system is hyperchaotic. In addition, the parameter interval of the 2D system in this paper that produces hyperchaos is very large. The above results show that the 2D system proposed in this paper has better chaotic properties.

### 2.4. Correlation Analysis

The correlation coefficient is an important indicator of the randomness of a time series. The autocorrelation coefficient and the cross-correlation of a time series with good randomness should be similar to the δ function and zero, respectively. The autocorrelation coefficient at lag k of a sequence $\left\{x(i),i=1,2,\ldots ,N\right\}$ of length N is shown in the following equation:

(5) $$autocorr\left(k\right)=\frac{\sum_{i=1}^{N-\left|\left.k\right|\right.}\left(x\left(i\right)-\bar{x}\right)\left(x\left(i+\left|\left.k\right|\right.\right)-\bar{x}\right)}{\sum_{i=1}^{N-\left|\left.k\right|\right.}{\left(x\left(i\right)-\bar{x}\right)}^{2}}$$

where $\bar{x}$ is the average value of the series $\left\{x(i)\right\}$.

The cross-correlation between two sequences $\left\{x(i)\right\}$ and $\left\{y(i)\right\}$, both of length N, is given by the following equation:

(6) $$crosscorr\left(k\right)=\frac{\sum_{i=1}^{N-\left|\left.k\right|\right.}\left(x\left(i\right)-\bar{x}\right)\left(y\left(i+\left|\left.k\right|\right.\right)-\bar{y}\right)}{\sqrt{\sum_{i=1}^{N-\left|\left.k\right|\right.}{\left(x\left(i\right)-\bar{x}\right)}^{2}}\sqrt{\sum_{i=1}^{N-\left|\left.k\right|\right.}{\left(y\left(i\right)-\bar{y}\right)}^{2}}}$$

where $\bar{x}$ and $\bar{y}$ are the averages of the $\left\{x(i)\right\}$ and $\left\{y(i)\right\}$ series, respectively.

**Figure 4:**
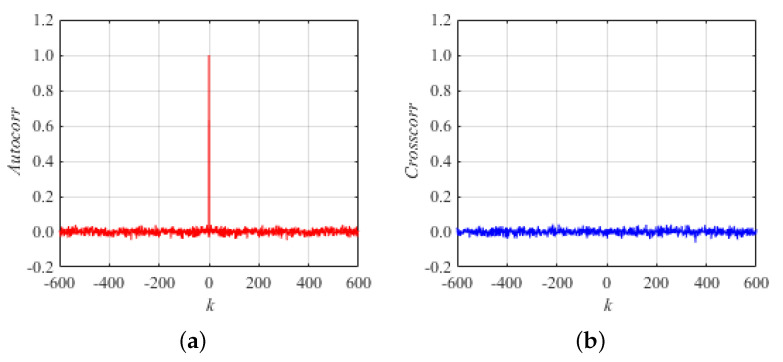
Plot of correlation for system (1): (**a**) autocorrelation; (**b**) cross-correlation.

For system (1), Figure 4a shows a plot of the autocorrelation coefficient of the random sequence $\left\{x(i)\right\}$ generated iteratively for the initial values of the system $\left(x_{0},y_{0}\right)=\left(0.299,0.674\right)$ and the control parameters a=10,b=20. Figure 4b shows the plot of the cross-correlation coefficient between two random sequences $\left\{x(i)\right\}$ and $\left\{y(i)\right\}$ generated iteratively for the initial state values of the system $\left(x_{0},y_{0}\right)=\left(0.299,0.674\right)$ and the control parameters a=10,b=20. In summary, the sequences generated by the new 2D map system proposed in this paper have good randomness, and their correlation and cross-correlation coefficient meet the requirements of random sequences.

### 2.5. Approximate Entropy Analysis

The approximate entropy (ApEn) of chaotic systems is a statistical tool to quantify the degree of chaos in a system and is used to measure the regularity and complexity of time series data. The core idea of approximate entropy is to compare the similarity of neighboring template vectors in a time series and to assess the complexity and unpredictability of the sequence by calculating the number of similar template pairs within a given tolerance. For a detailed step-by-step calculation of the approximate entropy, please refer to [23].

**Figure 5:**
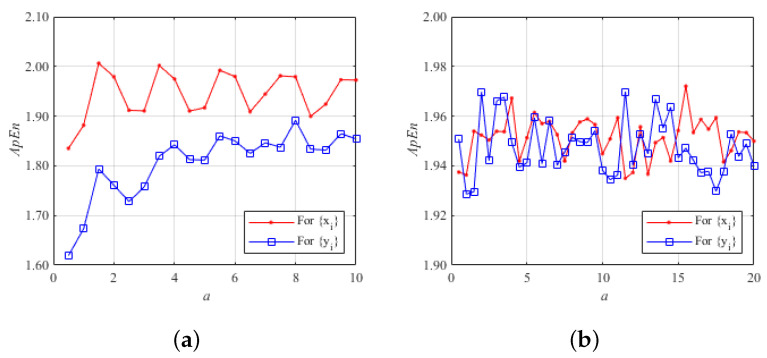
Approximate entropy of two different chaotic systems: (**a**) approximate entropy of the 2D-CLII chaotic system; (**b**) approximate entropy of the 2D chaotic system proposed in this paper.

Figure 5 shows a comparison of the approximate entropy values of two chaotic systems. Figure 5a plots the approximate entropy variation curves of the 2D-CLII chaotic system when the control parameter a is varied from 0 to 10. Figure 5b plots the approximate entropy variation curves of the 2D chaotic system proposed in this paper when the control parameter b=40 and a is varied in the range of 0 to 20.

As can be seen in Figure 5, the 2D chaotic system proposed in this paper has a larger approximation entropy and a wider range of parameter variations compared to the recently proposed 2D-CLII system. For system (1) in this paper, the average ApEn value of the generated sequences $\left\{x(i)\right\}$ is 1.9514 and the average ApEn value of the sequences $\left\{y(i)\right\}$ is 1.9474, while for the recently proposed 2D-CLII system, the average ApEn value of the generated sequences $\left\{x(i)\right\}$ is 1.9442 and the average ApEn value of the sequences $\left\{y(i)\right\}$ is 1.8057. It can also be seen from Figure 5 that the approximate entropy change of the 2D chaotic system proposed in this paper is more stable. Therefore, the proposed 2D chaotic system has better chaotic performance.

### 2.6. Permutation Entropy Analysis

The permutation entropy (PeEn) for chaotic systems is a statistical measure used to quantify the dynamic complexity of a time series, which captures the stochastic character of the sequence by calculating the pattern of arrangement and frequency of occurrence of values in the sequence. The more complex and stochastic the time series, the larger the PeEn value. Thus, it can be better applied in image encryption.

**Figure 6:**
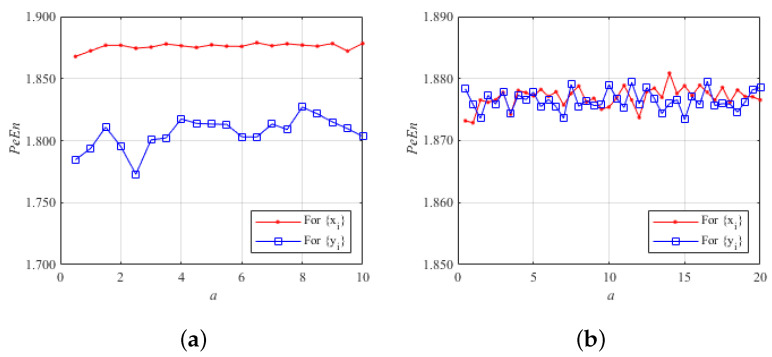
Permutation entropy of two different chaotic systems: (**a**) permutation entropy of the 2D-CLII chaotic system; (**b**) permutation entropy of the 2D chaotic system proposed in this paper.

Figure 6 shows the comparison of the PeEn values of two different 2D chaotic systems. Figure 6a shows the PeEn volatility of the 2D-CLII system with the variation of the system parameter *a* ranging from 0 to 10. The average PeEn value of the random sequence $\left\{x(i)\right\}$ generated iteratively by the 2D-CLII system is 1.8759, and the average PeEn value of the random sequence $\left\{y(i)\right\}$ is 1.8061. Figure 6b shows the PeEn volatility of the 2D chaotic system proposed in this paper with the parameter b=40, and the variation range of parameter *a* is from 0 to 20. The average PeEn value of the random sequence $\left\{x(i)\right\}$ generated by the 2D chaotic system proposed in this paper is 1.8770, and the average PeEn value of the random sequence $\left\{y(i)\right\}$ is 1.8765. From Figure 6, it can be seen that the fluctuation ranges of the PeEn values of the 2D chaotic system proposed in this paper are much smaller and the average value is higher than that of the 2D-CLII system. So, the 2D chaotic system proposed in this paper has a smaller range of fluctuation of the PeEn value, and the average value is higher than the 2D-CLII system. Therefore, the 2D chaotic system proposed in this paper can generate random sequences more stably and its chaotic performance is better.

### 2.7. NIST Test of the New Hyperchaotic Map

The NIST (National Institute of Standards and Technology, Gaithersburg, MD, USA) SP 800-22 test suite is widely used in fields such as cryptography, computer security, and statistical analysis to ensure that sequences generated by random number generators are sufficiently random and unpredictable. This test suite requires the provision of a large number of time sequences generated by a chaotic system for testing, each of which is 1,000,000 bits in length. There are two performance metrics, the *p*-value and pass rate, used to measure the stochastic performance of the time series. The default significance level α=0.01. The confidence interval used to test the pass rate is defined as $1-\alpha \pm 3\sqrt{\alpha (1-\alpha )/m}$, where *m* is the number of bit sequence groups. When α=0.01 and m=100, the confidence interval is $1-0.01\pm 3\sqrt{0.01\times 0.99/100}=\left[0.96,1.02\right]$, which indicates that the minimum pass rate must be 96%.

To test the random performance of the chaotic sequences generated by the proposed 2D hyperchaotic system, we generated 100 sequences of chaotic real numbers, each of which has a length of 1,000,000/8 real numbers. Since the NIST test software SP 800-22 requires a binary file for testing, we transformed the chaotic sequence into a binary file by Algorithm 1. The parameters were set to $a=20,b=30,x_{0}=0.123987$, and $y_{0}=0.987321$. The randomness of the bit sequences was then tested using the NIST software SP 800-22 package. The results of the NIST statistical test are shown in Table 1. As can be seen from the test results, each *p*-value is greater than 0.01, and the minimum *p*-value is 0.011931. Each pass rate is greater than 96%, and the minimum pass rate for each statistical test is 96%.
**Algorithm 1** Chaotic key stream-generation algorithm.**Input**: Chaotic sequence *X*. **Output**: The binary sequence file f55.bin. 1:$xb=mod(floor\left(X*10^{15}\right),256)$;   ▹ Get a sequence of integers with values 0–2552:FID = fopen(‘D:\NIST\f55.bin’,‘w’);3:COUNT = fwrite(FID,xb,‘uint8’);        ▹ Save the integer sequence xb as binary4:fclose(FID);

## 3. Image Compression and Reconstruction Algorithm

In the practical application scenarios of image encryption, there are often objective constraints such as limited channel transmission bandwidth and storage space. Therefore, how to make the encrypted ciphertext image occupy less space in order to reduce the communication pressure on the transmission channel is a topic worth studying. Both image encryption and compression need to be considered in such application scenarios; therefore, the study of new algorithms for joint image compression and encryption is also an issue to be explored in this paper. Combining compressed-sensing algorithms with chaotic encryption techniques can achieve simultaneous compression and encryption of plaintext images to effectively reduce the pressure of data transmission, storage, and processing. In the image-encryption algorithm based on compressed-sensing and chaotic systems, the chaotic system is mainly used to generate the encrypted key stream and construct the measurement matrix satisfying the finite isometric property.

### 3.1. An Overview of Compressed Sensing Theory

Compressed sensing (CS) is a breakthrough in the field of signal processing, which is a new signal-acquisition paradigm that can efficiently capture and recover the original signal through a set of small linear, non-adaptive samples. CS is initially used to sample one-dimensional signals; in order to apply CS to the compressed sampling of two-dimensional images, a two-dimensional image *X* of size N×N needs to be converted into a one-dimensional vector *x* of length $N^{2}\times 1$, and then, the one-dimensional vector *x* is compressed and sampled using 1DCS, which can be expressed as follows:

(7) y=Φx

where $x\in R^{N^{2}\times 1}$ is a one-dimensional column vector of length $N^{2}\times 1$, $\Phi \in R^{M^{2}\times N^{2}}$ is a measurement matrix of size $M^{2}\times N^{2}$, $y\in R^{M^{2}\times 1}$ is a one-dimensional column vector of length $M^{2}\times 1$, and y is referred to as the vector of measurements for x.

The CS sampling operator shaped as (7) is called one-dimensional CS (1DCS), and 1DCS requires $O(M^{2}N^{2})$ arithmetic operations and $M^{2}N^{2}$ memory cells to store the measurement matrix. Therefore, when 1DCS is used to sample 2D images, it faces two main challenges as follows [24]: First, the computational complexity of the codec is very high, and the time complexity of 1DCS is $O(M^{2}N^{2})$ operations. Secondly, the measurement matrix requires a large amount of storage space ($M^{2}N^{2}$ memory cells are required for storing the measurement matrix).

In order to compensate for the above drawbacks of one-dimensional compressed sensing for compressed sampling of two-dimensional image information, two-dimensional compressed sensing (2DCS) has been proposed. Assuming that $\Phi_{1}$ and $\Phi_{2}$ are both random matrices of size M×N(M≪N), the 2D measurements $Y\in R^{M\times M}$ of an image X of size N×N can be obtained by (8).

(8) $$Y=\Phi_{1}X{\Phi_{2}}^{T}$$

where $\Phi_{1}$ and $\Phi_{2}$ are called the measurement matrices, $\Phi_{1}$ operates on the rows of the 2D image, while $\Phi_{2}$ operates on its columns. Compared to 1DCS, 2DCS requires only the time overhead of $O(MN^{2})$ operations and 2MN memory cells for storing the measurement matrix. Thus, 2DCS significantly reduces the computational complexity of the codec and saves storage space for the random measurement matrix, making this sampling operator more suitable for sampling 2D images.

Since the size of Y is much smaller than the size of X, Y is considered as a downscaled version of X and the sampling rate (SR) is defined as:

(9) $$SR=\frac{M^{2}}{N^{2}}$$

It should be noted that CS and data compression serve different purposes. CS aims to reduce the number of samples, which are usually represented as real values with infinite precision. In contrast, data compression focuses on reducing the number of bits occupied by information. Even though CS can significantly reduce the number of samples, the bit width occupied by each sample store may increase. Typically, for an image with 256 levels of grey scale, an 8-bit representation is required for each pixel. However, after CS encoding, the number of bits required to represent each CS real-valued sample increases significantly. In order to reduce the number of bits for transmitting the information, it is necessary to perform the quantization of the values of the CS real-valued samples, which is to map the values of the CS real-valued samples to a range of pixel values of a 256-level grey-scale image, i.e., integers in the interval [0,255].

The process of recovering the original image information from the compression-aware measurements of an image is called image reconstruction, which is the inverse process of compression-aware compressive sampling. In practice, many reconstruction algorithms have been proposed for 2DCS, such as the 2D projected gradient (2DPG) algorithm [19], the 2D orthogonal matching tracking (2D-OMP) algorithm [25], and the 2D smoothing L0 (2D-SL0) [26] algorithm, which are all effective at reconstructing 2D compressed perceptual information.

In this paper, the 2D projected gradient (2DPG) algorithm will be used to achieve the reconstruction of the original image from the 2D perceptual measurements of the image. The 2DPG algorithm used in this paper to reconstruct the compressed image is a modified version of the open-source code of the 2DPG-ED algorithm in Reference [24].

### 3.2. Image Compression Algorithm

Since 2DCS has better performance than 1DCS, this paper uses 2DCS for image compression. Since the dynamic range of the compressed sample signals output from the 2DCS module affects the final compression performance, if the dynamic range of the compressed samples can be reduced by using certain compression strategies, fewer bits can be used to represent each compressed sample, thus improving the compression performance. Therefore, the image compression process in this paper also adopts a grey scale mapping strategy to perform the uniform translation of the pixel values of the original image, i.e., B=A−F (the pixel values of A are uniformly shifted downward by an amplitude of 128 to obtain B); the grey-scale mapped image information B is then input into the 2DCS processing module to reduce the dynamic range of the compressed samples Y, so as to improve the final compression performance. In addition, since the effect of image reconstruction is not only related to the 2DCS reconstruction algorithm, but also related to the random performance of the measurement matrix, this paper adopts a chaotic system to generate the two measurement matrices required for 2DCS compression sampling $\Phi_{1}$ and $\Phi_{2}$. For practical image-compression systems, it is necessary to use some quantization strategies to generate a compressed bitstream of CS samples. In order to make the quantized information consistent with the range of pixel values of common digital images, in this paper, the values of CS real-valued samples are quantized into integers in the interval [0, 255] by mapping.

The algorithmic steps of the image compression process can be described in detail as follows: Step 1: In grey scale mapping, the pixel values of the original image are uniformly shifted using Equation (10):

(10) B=A−F

where F is an N×N constant matrix with element values of 128 (*N* is the number of rows or columns of the original image).

Step 2: Generate two measurement matrices $\Phi_{1}$ and $\Phi_{2}$ using chaotic mapping.

In this paper, system (1) is used to generate two random matrices U and V. The specific generation algorithm is shown in Algorithm 2.
**Algorithm 2** Generating measurement matrices using 2D hyperchaotic map.**Input**: Parameters of the hyperchaotic system a,b; initial values of the system state $x_{0},y_{0}$; number of rows of the image matrix: *N*; compressed sensing parameter: subrate. **Output**: Two measurement matrices: $\Phi_{1}$ and $\Phi_{2}$. 1:Initialize two one-dimensional arrays U and V of size N×N;2:Let $U\left(1\right)=x_{0}$ and $V\left(1\right)=y_{0}$;3:**for** i=2:N×N **do**4:   $U\left(i\right)=\sin^{2}\left(a\pi U(i-1)+bV(i-1)\right)$;5:   $V\left(i\right)=\cos^{2}\left(b\pi /V(i-1)+aU(i-1)\right)$;6:**end for**7:U=reshape(U,N,N);8:V=reshape(V,N,N);9:$M=round(\sqrt{subrate}\times N)$;10:$\Phi_{1}=orth\left(U\right)$; $\Phi_{1}=\Phi_{1}\left(1:M,:\right)$;11:$\Phi_{2}=orth\left(V\right)$; $\Phi_{2}=\Phi_{2}\left(1:M,:\right)$;

Then, the standard orthogonal bases $\Phi_{1}$ and $\Phi_{2}$ applicable to the ranges of U and V are obtained from the random matrices U and V, respectively:

(11) $$\Phi_{1}=orth\left(U\right),\Phi_{2}=orth\left(V\right)$$

Here, the function orth(U) serves to return a standard orthogonal basis applicable to the range of U. Finally, the first *M* rows are taken for $\Phi_{1}$ and $\Phi_{2}$ to obtain new submatrices $\Phi_{1}=\Phi_{1}\left(1:M,:\right)$ and $\Phi_{2}=\Phi_{2}\left(1:M,:\right)$ as the 2D compressed perception measurement matrices, where $M=\sqrt{CR}\times N\ll N$ and SR is the sampling rate defined by Equation (9).

Step 3: 2DCS compression sampling is performed on the grey scale mapped image B to obtain the sampled measurement value matrix Y. The specific algorithm is shown in the 2DCS key Equation (12):

(12) $$Y=\Phi_{1}B{\Phi_{2}}^{T}=\Phi_{1}\left(A-F\right){\Phi_{2}}^{T}=\Phi_{1}A{\Phi_{2}}^{T}-\Phi_{1}F{\Phi_{2}}^{T}=A_{\mathrm{cs}}-F_{\mathrm{cs}}$$

where $A_{\mathrm{cs}}=\Phi_{1}A{\Phi_{2}}^{T}$ and $F_{\mathrm{cs}}=\Phi_{1}F{\Phi_{2}}^{T}$ are the compression perception measurements of A and F, respectively.

Step 4: The quantization of the matrix of sampled measurements Y yields the output compressed image of the session Z. The algorithm for quantization is shown in the following Equation (13):

(13) $$Y_{\max}=\max\left(\max\left(Y\right)\right),Y_{\min}=\min\left(\min\left(Y\right)\right),Z=\left[\frac{(Y-Y_{\min})\times 255}{\left(Y_{max}-Y_{\min}\right)}\right]$$

where max() and min() are the maximum and minimum functions, respectively, and [x] is rounding to the nearest integer of *x*.

### 3.3. Image Reconstruction Algorithm

At the decoding end, the original image can be reconstructed from the compressed image Z by four sub-steps. Firstly, the measurement matrices $\Phi_{1}$ and $\Phi_{2}$ are generated from the chaotic mapping and its parameters. Secondly, the two-dimensional measurements Y are recovered by using the inverse quantization process (the inverse quantization does not recover Y completely and accurately due to the irreversibility of the rounding operation in the quantization step, so the recovered Y can be expressed as Y′). Third, $F_{cs}$ can be derived from $\Phi_{1}$ and $\Phi_{2}$, and thus, $A_{cs}$ can be recovered (the recovered $A_{cs}$ can be expressed as ${A^{\prime }}_{cs}$). Finally, the original image is recovered from ${A^{\prime }}_{cs}$ using the improved 2DPG algorithm in this paper to obtain the reconstructed image $A^{\prime }$. The detailed description of the steps of the image-reconstruction process is as follows:

Step 1: Generate the measurement matrices $\Phi_{1}$ and $\Phi_{2}$ from the chaotic mapping and its parameters, with the same algorithm as before (omitted here).

Step 2: The inverse quantization recovers a two-dimensional measurement Y, denoted by $Y^{\prime }$, which is calculated as:

(14) $$Y^{\prime }=\left(Y_{\max}-Y_{\min}\right)\times Z/255+Y_{\min}$$

Step 3: Recover $A_{cs}$ (denoted by ${A^{\prime }}_{cs}$), which is calculated as follows:

(15) $$F_{\mathrm{cs}}=\Phi_{1}F{\Phi_{2}}^{T}$$

(16) $${A^{\prime }}_{cs}=Y^{\prime }+F_{cs}$$

Step 4: Based on $A_{\mathrm{cs}}=\Phi_{1}A{\Phi_{2}}^{T}$, invoke the improved 2DPG algorithm to reconstruct the original image A from the recovered measurements ${A^{\prime }}_{cs}$ (the reconstructed A is denoted by A′).

## 4. The Proposed Image Encryption and Decryption Scheme

For the ease of reading and understanding, this article provides some explanations of some of the symbols and functions that appear in encryption and decryption algorithms (Table 2).

### 4.1. Encryption Algorithm

The image-encryption algorithm proposed in this paper combines chaotic key sequence generation, image pixel disruption, and image pixel diffusion simultaneously.

The secret key for the encryption scheme proposed in this paper consists of five double-precision floating-point numbers $\left\{x_{0},y_{0},a,b,k\right\}$ and an 8-bit unsigned integer Pre. The four double-precision floating-point numbers $\left\{x_{0},y_{0},a,b\right\}$ serve as the iterative initial values for the 2D hyperchaotic system, while the 8-bit unsigned integer Pre and double-precision floating-point number *k* are used as intermediate keys for encryption. The image-encryption algorithm consists of two main parts: chaotic key sequence generation, synchronized pixel disruption, and diffusion. These two main parts alternate to closely associate the key with the image pixel values, which improves the difficulty of key cracking. This greatly improves the security of the encryption algorithm. The specific steps of the encryption algorithm are as follows (Algorithm 3).
**Algorithm 3** Image-encryption algorithm.**Input**: Parameters of the hyperchaotic system $\left\{x_{0},y_{0},a,b\right\}$, plaintext image **P**, the initial values of the intermediate key Pre and *k*. **Output**: Ciphertext image **C** and the secret key sequences {X,Y}. 1:[M,N]=size(P);                                                 ▹ Get image size2:flag=zeros(M,N);                                          ▹ Initialize a zero matrix flag3:X=zeros(1,M∗N);4:Y=zeros(1,M∗N);                                   ▹ Initialize the secret key sequences {X,Y}5:**for** i=1:M **do**6:   **for** j=1:N **do**7:   Random values {x,y} are obtained iteratively using system (1).8:   $i_{next}=mod\left(floor\left(x*10^{6}\right),M\right)+1$;9:   $j_{next}=mod\left(floor\left(y*10^{6}\right),N\right)+1$;10:    **while** $flag(i_{next},j_{next})==1$ **do**11:    Repeat steps 7 to 9;12:    **end while**13:    $C\left(i_{next},j_{next}\right)=bitxor\left(mod\left(P\left(i,j\right)+k,256\right),Pre\right)$;14:    $Pre=C(i_{next},j_{next})$;15:    $flag(i_{next},j_{next})=1$;16:    Record {x,y} to sequences {X,Y}, respectively.17:   **end for**18:**end for**

### 4.2. Decryption Algorithm

The image decryption algorithm is the inverse of the image encryption algorithm, as shown below (Algorithm 4).
**Algorithm 4** Image-decryption algorithm.**Input**: Ciphertext image **C**, the secret key sequences {X,Y}, the initial values of the intermediate key Pre and *k*. **Output**: Plaintext image **P**. 1:[M,N]=size(P);                                 ▹ Get image size2:num=1;                        ▹ Position number of the secret key sequence3:**for** i=1:M **do**4:   **for** j=1:N **do**5:     $i_{next}=mod\left(floor\left(X\left(num\right)*10^{6}\right),M\right)+1$;6:     $j_{next}=mod\left(floor\left(Y\left(num\right)*10^{6}\right),N\right)+1$;7:     $P\left(i,j\right)=mod(bitxor\left(C\left(i_{next},j_{next}\right),Pre\right)-k,256)$;8:     $Pre=C(i_{next},j_{next})$;9:     num=num+1;10:   **end for**11:**end for**

## 5. Experimental Results and Security Analysis

We used MATLAB 2022b to verify the performance of the image-compression-recon- struction and image-encryption algorithms on a computer with an Intel(R) Core(TM) i5-10300H @ 2.50 GHz CPU and 16.0 GB of RAM. The test images were obtained from USC-SIPI.

### 5.1. Image Compression and Reconstruction Performance Analysis

Classical 512 × 512 grey scale images (N = 512) including Lena, Barbara, peppers, and cameraman were used for the experiments. The parameters of the experiments were set as follows: the parameters of the 2D hyperchaotic system used to generate the two measurement matrices were $\left\{a=20,b=30,x_{0}=0.128,and\;y_{0}=0.982\right\}$; as a comparison experiment, the parameters to generate the Logistic chaotic system for the two measurement matrices were $\left\{\mu =4,x_{0}=0.17\;or\;0.27\right\}$; the sampling rate SR=0.5; the parameters in the improved 2DPG algorithm were set as follows: the number of double-tree Discrete Wavelet Transform (DDWT) levels was 3; λ=6; the maximum number of iterations was 200; the error tolerance value was 0.000001.

The experiments will compare the difference in the quality of compressed reconstructed images between the new model in this paper and the classical Logistic chaos system generating the measurement matrix. Here, the recovery quality of the reconstructed image is measured by calculating two metrics, Peak signal-to-noise ratio (PSNR) and Structural Similarity (SSIM), between the reconstructed image and the original image, and the larger the value of the two metrics, the better the quality of the reconstructed image is. The PSNR is a measure of image quality, especially when evaluating image-compression or image-restoration algorithms. The PSNR is calculated based on the maximum possible power of the signal, which correlates with the signal-to-noise ratio (SNR) of the image. The PSNR is calculated using the following formula:

(17) $$PSNR=10\times \log\left(\frac{255^{2}}{MSE}\right)$$

where 255 is the maximum possible pixel value of the image; for an 8-bit image this is usually 255. MSE is the Mean Squared Error (MSE) and is calculated as:

(18) $$MSE=\frac{1}{M\times N}\sum_{i=1}^{M}\sum_{j=1}^{N}{\left(I_{i,j}-K_{i,j}\right)}^{2}$$

Here, I is the original image, K is the processed image, and M and N are the number of rows and columns of the image, respectively.

The SSIM is a more complex image-quality-evaluation index, which not only considers the brightness and contrast of the image, but also the structural information of the image. The calculation formula of the SSIM is as follows:

(19) $$SSIM\left(x,y\right)=\frac{\left(2\mu_{x}\mu_{y}+c_{1}\right)\left(2\sigma_{xy}+c_{2}\right)}{\left(\mu_{x}^{2}+\mu_{y}^{2}+c_{1}\right)\left(\sigma_{x}^{2}+\sigma_{y}^{2}+c_{2}\right)}$$

where x and y are the original and processed images, respectively; $\mu_{x}$ and $\mu_{y}$ are the means of the images; $\sigma_{x}^{2}$ and $\sigma_{y}^{2}$ are the variances of the images; $\sigma_{xy}$ is the covariance of the images x and y; and $c_{1}$ and $c_{2}$ are small constants used to avoid having a denominator of zero.

**Figure 7:**
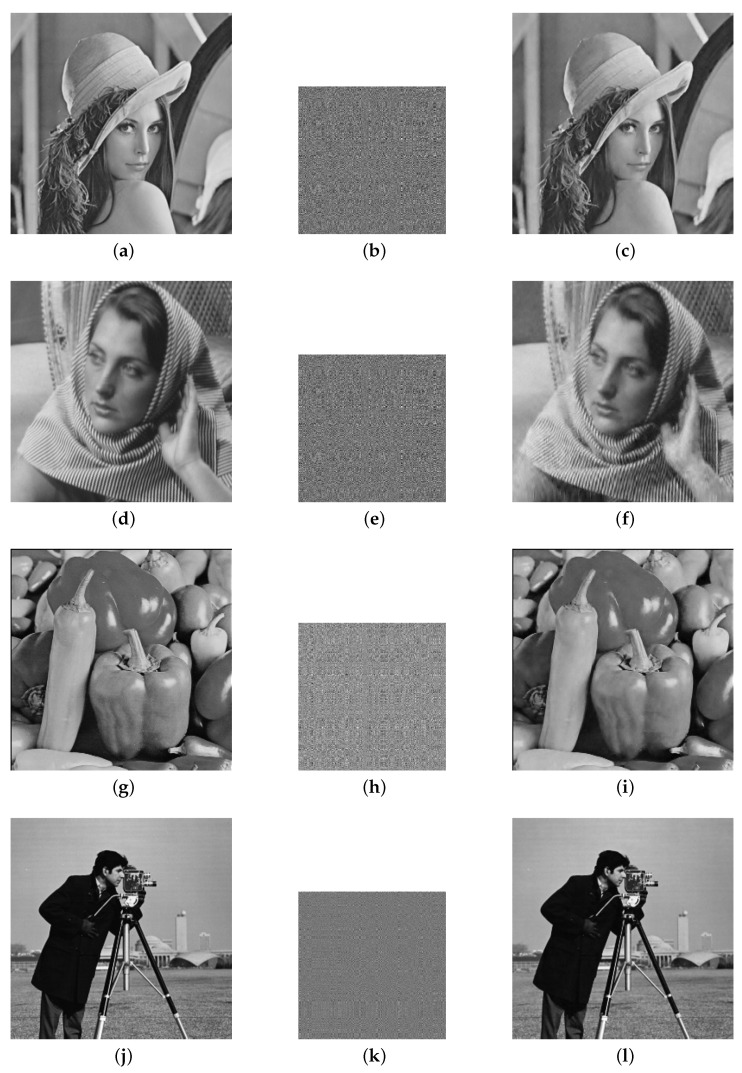
Four classical test images and their compressed sampled and reconstructed images: (**a**) original image Lena, (**b**) compressed and encrypted image Lena, (**c**) reconstructed image Lena, (**d**) original image Barbara, (**e**) compressed and encrypted image Barbara, (**f**) reconstructed image Barbara, (**g**) original image peppers, (**h**) compressed and encrypted image peppers, (**i**) reconstructed image peppers, (**j**) original image cameraman, (**k**) compressed and encrypted image cameraman, and (**l**) reconstructed image cameraman.

Figure 7 shows the intuitive results of the above test images, compressed images, and reconstructed images obtained by using the new 2D chaotic system; the human eye basically cannot distinguish the difference between the reconstructed image and the original image; when the sampling rate SR=0.5, M=362, so the size of the compressed sampled image is 362 × 362.

Table 3 exhibits a comparison of the reconstructed image quality results using the chaotic system (1) and the Logistic chaotic system to generate the measurement matrix. The PSNR and SSIM values of the reconstructed images show that the reconstructed images obtained by using the new 2D chaotic system for generating the random measurement matrices were of better quality than those obtained by using the Logistic chaotic system for generating the random measurement matrices.

### 5.2. Histogram Analysis of Pixel Distribution

**Figure 8:**
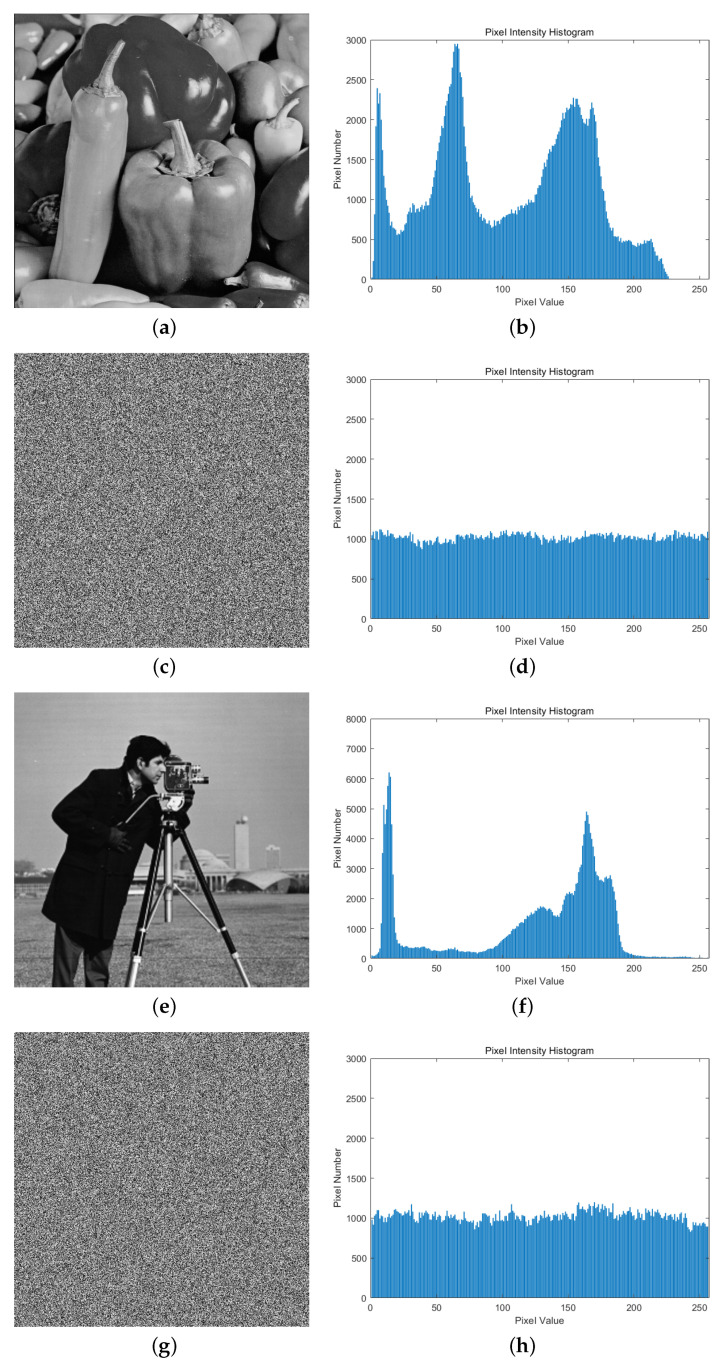
Histogram of pixel distribution of plaintext image and ciphertext image of two test images. (**a**) Plaintext image peppers. (**b**) Histogram of plaintext image peppers. (**c**) Ciphertext image peppers. (**d**) Histogram of ciphertext image peppers. (**e**) Plaintext image cameraman. (**f**) Histogram of plaintext image cameraman. (**g**) Ciphertext image cameraman. (**h**) Histogram of Ciphertext image cameraman.

The distribution of image pixel values can be visualized by the pixel histogram, and the pixels of a plaintext image with actual semantics should have a strong correlation, which is usually shown in the histogram as a concentrated distribution towards a few pixel values. However, the image-encryption algorithm should break this strong correlation of the pixels of a plaintext image, so that the histogram of the pixels of the encrypted image shows a relatively flat distribution, making it impossible for an attacker to infer the original plaintext image information by counting the distribution of the pixel values. Figure 8 shows the comparison of the histograms of pixel distributions before and after the encryption of the two test images, from which it can be seen that our proposed image-encryption algorithm breaks the correlation between the plaintext image pixels and has a good ability to resist the statistical pixel value attack.

### 5.3. Secret Key Space Size Analysis

The secret key of our proposed image-encryption algorithm consists of five double-precision floating-point numbers and one 8-bit unsigned integer, namely $\left\{x_{0},y_{0},a,b,k,Pre\right\}$. Since each double-precision floating-point number accounts for 64 bits, the five double-precision floating-point numbers account for a total of 320 bits. An 8-bit unsigned integer occupies 8 bits, so the size of the key space of our proposed algorithm is about $2^{328}$. According to the National Institute of Standards and Technology (NIST) literature [27], the cryptographic algorithm is proven to be effective against brute-force attacks when the key space is greater than $2^{128}$. Therefore, our proposed image-encryption algorithm satisfies the above requirement.

### 5.4. Information Entropy Analysis of Encrypted Images

In the field of image encryption, the concept of Shannon’s entropy of information is used to assess the randomness and complexity of an image, so it is usually used to evaluate the security of image-encryption algorithms. The ideal value of the information entropy of a completely random image is 8, so the closer the information entropy of the encrypted image is to 8, the more secure the encryption algorithm is. Assuming a source of information as x, the information entropy formula is shown below:

(20) $$H\left(x\right)=-\sum_{i=1}^{L}P(x_{i})\log_{2}\left[P(x_{i})\right]$$

Table 4 shows the information entropy of the encrypted image obtained by our proposed scheme in comparison with other references, from which it can be seen that the information entropy of the encrypted image obtained by our scheme is closer to the ideal value of 8.

### 5.5. Image Adjacent Pixel Correlation Analysis

In general, a plaintext image with actual semantics has a strong correlation between adjacent pixel pairs, as evidenced by the fact that their pixel values are relatively close to each other. However, a good image-encryption algorithm should break this correlation so that there is no correlation between adjacent pixel pairs of an encrypted image. In this way, an attacker will not be able to obtain any useful information from the adjacent pixel pairs of an encrypted image that can decipher the ciphertext. The correlation coefficient (CC) between adjacent pixel pairs in an image is generally calculated using the following formula:

(21) $$CC\left(a,b\right)=\frac{\sum_{i=1}^{N}\left(a_{i}-\bar{a}\right)\left(b_{i}-\bar{b}\right)}{\sqrt{\sum_{i=1}^{N}{\left(a_{i}-\bar{a}\right)}^{2}\sum_{i=1}^{N}{\left(b_{i}-\bar{b}\right)}^{2}}}$$

where $a=\{a_{1},a_{2},\ldots ,a_{N}\}$ and $b=\{b_{1},b_{2},\ldots ,b_{N}\}$ are adjacent pixel pairs in an image. For example, $a_{i}$ and $b_{i}$ are adjacent. N is the number of adjacent pixel pairs selected in total. $\bar{a}$ and $\bar{b}$ are the average of the *a* and *b* pixel values, respectively. We randomly selected a certain number of adjacent pixel pairs by the horizontal, vertical, and diagonal directions, and the results of the calculated correlation coefficients are shown in Table 5. It can be seen from the results in the table that, in most cases, the CC value of the algorithm in this paper is closer to 0. This shows that the image-encryption algorithm in this paper has some advantages compared with the existing 2D chaotic system image-encryption algorithms.

## 6. Conclusions

In this paper, a new 2D discrete hyperchaotic system is proposed, and the chaotic performance of this hyperchaotic system is verified by bifurcation diagrams, Lyapunov diagrams, approximate entropy, and permutation entropy. The new 2D hyperchaotic system as a pseudo-random number generator can completely pass all the test items of NIST and is suitable for constructing measurement matrices for 2D compressed perception and generating key sequences for image encryption. Compared with some existing 2D chaotic systems, it enhances the complexity of the system and possesses good chaotic performance, which improves the security of encryption. In addition, in this paper, the image is encrypted after compression using two-dimensional compressed sensing (2DCS), which effectively reduces the amount of data transmission of the encrypted image. In addition, the existing 2D projected gradient (2DPG) algorithm is improved to enhance the quality of image reconstruction. Finally, this paper designs an encryption algorithm for simultaneous disruption and diffusion of image pixels for the new 2D chaotic system. The security of the algorithm was verified by experiments such as key space size analysis and information entropy comparison of encrypted images.

Although the image-compression-measurement matrix generated by the new 2D chaotic map designed in this paper can achieve better image reconstruction than the traditional chaotic map, it still does not change the nature of lossy image compression, and the compression performance of the image is still greatly affected by the measurement matrix. In the future, the powerful learning ability of deep neural networks can be explored and used to compress images to achieve the purpose of lossless image compression.

## Figures and Tables

**Table 1:** NIST statistical test results for the 2D hyperchaotic system proposed in this paper.

Name of Test Item	*p*-Value	Pass Rate	Results
Frequency	0.616305	100/100	pass
Block Frequency	0.978072	99/100	pass
Cumulative Sums (Forward)	0.779188	100/100	pass
Cumulative Sums (Reverse)	0.798139	100/100	pass
Runs	0.834308	99/100	pass
Longest Run	0.030806	100/100	pass
Rank	0.554420	99/100	pass
FFT	0.019188	100/100	pass
Non-Overlapping Template *	0.129620	96/100	pass
Overlapping Template	0.090936	99/100	pass
Universal	0.437274	99/100	pass
Approximate Entropy	0.289667	99/100	pass
Random Excursions *	0.264458	59/61	pass
Random Excursions Variant *	0.011931	59/61	pass
Serial Test 1	0.025193	99/100	pass
Serial Test 2	0.883171	100/100	pass
Linear Complexity	0.798139	100/100	pass

* Note: Non-Overlapping Template, Random Excursions, and Random Excursions Variant all contain multiple sub-tests, and the worst results among the sub-tests are given in Table 1.

**Table 2:** Explanation of some symbols.

Symbol	Meaning	Property
**P**	Indicates the plaintext image to be encrypted	Two-dimensional (2D) matrix
**C**	Indicates a ciphertext image that has been encrypted	Two-dimensional (2D) matrix
$\left\{x_{0},y_{0},a,b\right\}$	2D hyperchaotic system parameters	Double-precision floating-point numbers
Pre	Intermediate secret key	8-bit unsigned integer
*k*	Intermediate secret key	Double-precision floating-point number

**Table 3:** Comparison of reconstructed image quality metrics of test images obtained using different chaotic systems.

Test Metrics	Lena	Barbara	Peppers	Cameraman
PSNR (system (1))	**36.4229**	**32.0886**	**36.2467**	**37.3994**
SSIM (system (1))	**0.91895**	**0.89765**	**0.90834**	**0.93256**
PSNR (Logistic system)	35.6712	31.7618	35.6433	37.1623
SSIM (Logistic system)	0.9050	0.88843	0.89699	0.92974

Note: The results of the system in this paper are shown in bold.

**Table 4:** Comparison of information entropy of encrypted images obtained by different encryption algorithms.

Test Images	Plain Image	Ours	Ref. [28]	Ref. [29]	Ref. [30]
Lena	7.4456	7.9989	7.9977	7.9970	7.9914
Baboon	7.3579	7.9992	\	\	7.9917
Peppers	7.5715	7.9987	7.9976	7.9971	7.9915
Cameraman	7.0480	7.9968	\	7.9971	\

**Table 5:** Comparison results of the CC values of the algorithm proposed in this paper with other similar algorithms (image: Lena).

Image-Encryption Algorithm	Horizontal	Vertical	Diagonal
Original image	0.97189	0.98498	0.95928
**Ours**	**−0.00031181**	**−0.00037405**	**0.00015204**
Ref. [31]	− 0.00062	0.0022	−0.0015
Ref. [32]	0.024095	−0.022246	0.016913
Ref. [33]	0.0062	−0.0001	0.0018
Ref. [34]	−0.0006	0.0010	−0.0012

Note: The results of the system in this paper are shown in bold.

## Data Availability

The data presented in this study are available on request from the corresponding author.
